# Glucosidase Inhibition to Study Calnexin-assisted Glycoprotein Folding in Cells

**DOI:** 10.21769/BioProtoc.3248

**Published:** 2019-06-05

**Authors:** Hao Wang, Qingyu Wu

**Affiliations:** 1 Department of Cardiology, Shanghai Institute of Cardiovascular Diseases, Zhongshan Hospital, Fudan University, Shanghai, China; 2 Cyrus Tang Hematology Center, Collaborative Innovation Center of Hematology, State Key Laboratory of Radiation Medicine and Prevention, Soochow University, Suzhou, China; 3 Department of Cardiovascular & Metabolic Sciences, Lerner Research Institute, Cleveland Clinic, Cleveland, USA

**Keywords:** Calnexin, Corin, α-Glucosidase, Glucosidase inhibition, Glycoprotein folding, N-glycosylation, Prothrombin

## Abstract

Calnexin is a chaperone protein that plays a critical role in glycoprotein folding in the endoplasmic reticulum (ER). The function of calnexin depends on its binding to monoglucosylated oligosaccharides on nascent glycoproteins, whereas the generation of monoglucosylated oligosaccharides depends on the activity of α-glucosidases I and II, which trim off terminal glucose residues sequentially from triglucosylated N-glycans. This biochemical mechanism can be exploited to study calnexin-assisted folding and subsequent ER exiting of glycoproteins in cells. In our investigation of the intracellular trafficking of N-glycosylated serine proteases, we used an inhibitor of α-glucosidases I and II to block the trimming of triglucosylated oligosaccharides, thereby inhibiting calnexin-assisted glycoprotein folding. The study helped us to discover a key role of calnexin in the folding, ER exiting, and extracellular expression of N-glycosylated serine proteases such as corin, enteropeptidase, and prothrombin. A similar approach of glucosidase inhibition can be used to study the calnexin/calreticulin-dependent folding and intracellular trafficking of other N-glycosylated proteins.

## Background

N-glycosylation is a common post-translational modification that occurs in approximately two thirds of the proteins encoded by the human genome (Apweiler *et al.*, 1999). Serine proteases of the trypsin fold are important in diverse physiological processes, such as hormone processing, blood coagulation, food digestion, cell differentiation, signal transduction, and tissue remodeling (Perona and Craik, 1995; Overall and Blobel, 2007; Antalis *et al.*, 2011; Zhou and Wu, 2014; Tanabe and List, 2017). In many of these proteases, N-glycosylation is essential for their intracellular trafficking, extracellular expression, and zymogen activation in different cell types (Bolt *et al.*, 2007; Liao *et al.*, 2007; Miyake *et al.*, 2010; Jiang *et al.*, 2014). To date, however, the biochemical mechanisms underlying the N-glycan-dependent processes are not fully understood.

Calnexin is a chaperone protein that acts as a key partner in the calnexin-calreticulin cycle to promote glycoprotein folding in the ER (Ellgaard and Frickel, 2003; Caramelo and Parodi, 2008; Lamriben *et al.*, 2016). Although calnexin can stabilize proteins via direct protein-protein interactions (Williams, 2006; Wang *et al.*, 2010), the function of clanexin in promoting glycoprotein folding requires its binding to monoglucosylated N-glycans on nascent proteins (Caramelo and Parodi, 2008; Lamriben *et al.*, 2016). This mechanism necessitates the activity of α-glucosidases I and II, which sequentially remove terminal glucose residues from triglucosylated N-glycans, making monoglucosylated oligosaccharides available for calnexin biding (Roth *et al.*, 2010).

Corin is a serine protease of the trypsin fold that regulates body fluid balance and cardiovascular homeostasis (Yan *et al.*, 2000; Cui *et al.*, 2012; Li *et al.*, 2017). In mice, corin deficiency causes hypertension and cardiac hypertrophy (Buckley and Stokes, 2011; Wang *et al.*, 2012; Baird *et al.*, 2019). Corin protein is heavily N-glycosylated. To date, no O-glycans and sialic acids have been detected in corin (Liao *et al.*, 2007). We and others have shown that N-glycosylation is critical for corin expression on the cell surface (Liao *et al.*, 2007; Gladysheva *et al.*, 2008), where zymogen corin is converted to an active enzyme by proprotein convertase subtilisin/kexin-6 (Chen *et al.*, 2015). Mutations abolishing N-glycosylation in the corin protease domain prevent corin activation (Wang *et al.*, 2015). Additional biochemical, cellular, and proteomic studies suggested a potential calnexin-corin interaction in the ER (Wang *et al.*, 2018).

**Figure 1.**
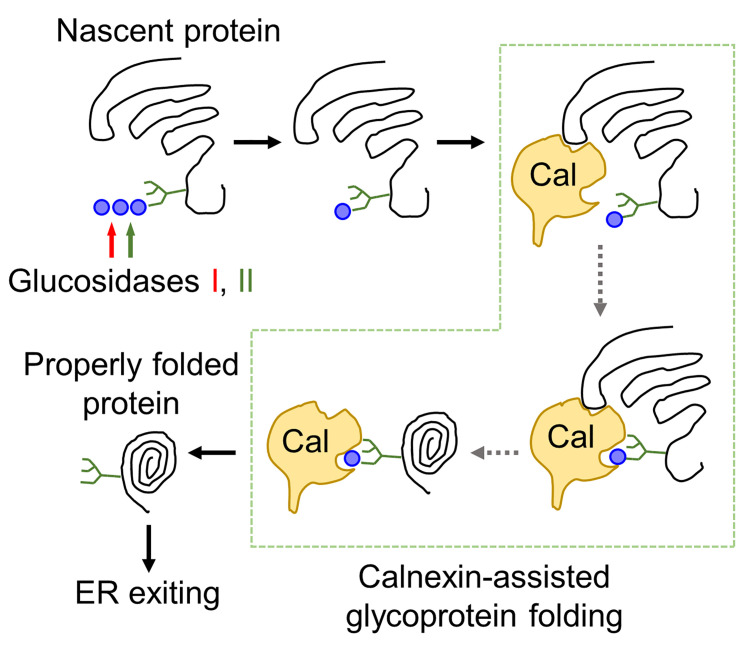
Illustration of calnexin-assisted glycoprotein folding in the ER. Terminal glucose residues (blue dots) of triglucosylated oligosaccharides on nascent proteins are trimmed by glucosidases I (red arrow) and II (green arrow) sequentially. Calnexin (Cal) binding to monoglucosylated N-glycans facilitates glycoprotein folding and subsequent ER exiting.

**Figure 2.**
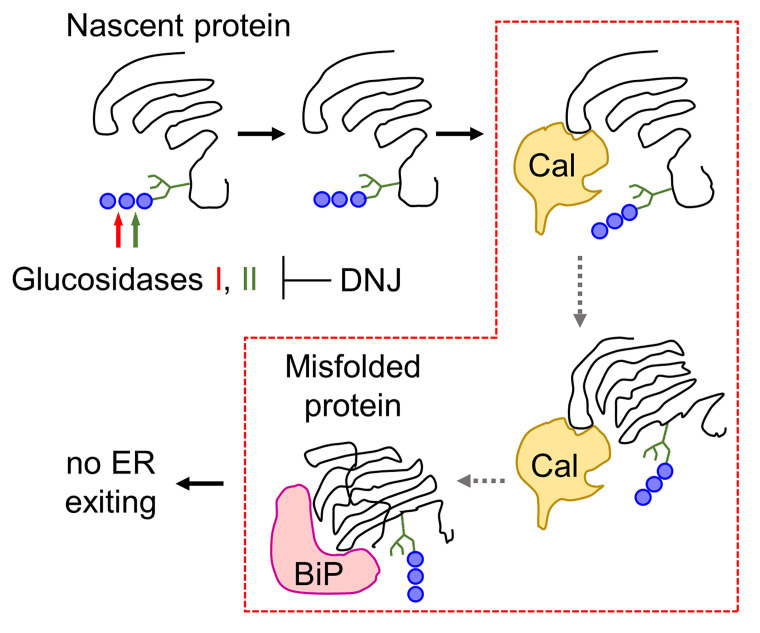
Glucosidase inhibition prevents calnexin-assisted glycoprotein folding. Inhibition of glucosidases I and II activity by 1-deoxynojirimycin (DNJ) prevents the trimming of triglucosylated oligosaccharides on nascent proteins, thereby blocking calnexin (Cal)-assisted glycoprotein folding. Misfolded proteins are retained in the ER by chaperone proteins, such as binding immunoglobulin protein (BiP), a key protein in the protein quality control system.

To examine the role of calnexin in the folding and ER exiting of N-glycosylated serine proteases, such as corin (a transmembrane protease) and prothrombin (a secreted protease), we conducted a study based on the underlying biochemical mechanism of calnexin-assisted glycoprotein folding, *i.e.*, the dependence of calnexin binding to monoglucosylated, but not triglucosylated, oligosaccharides on nascent proteins in the ER (Figure 1). We treated corin- and prothrombin-expressing cells with a natural compound, 1-deoxynojirimycin (DNJ), which inhibits α-glucosidases I and II, thereby preventing the generation of monoglucosylated oligosaccharides and hence the calnexin-N-glycan interaction (Saunier *et al.*, 1982; Gao *et al.*, 2016) (Figure 2). We then analyzed the ER chaperone protein binding and extracellular expression of corin and prothrombin. These studies helped us to discover a key role of calnexin, but not calreticulin, in assisting the folding and ER exiting of N-glycosylated serine proteases (Wang *et al.*, 2018). This glucosidase inhibition-based method can be used to study the folding and intracellular trafficking of other glycoproteins.

## Materials and Reagents

1. Pipette tips (any brand)
2. 1.5 ml microcentrifuge tubes (any brand)
3. 6-well cell culture plates (CytoOne, catalog number: CC7682-7506)
4. 100 mm cell culture dishes (Corning, catalog number: 430167)
5. HEK293 cells (ATCC, catalog number: CRL-1573)
6. Dulbecco’s Modified Eagle’s medium (Lerner Research Institute Cell Culture Core, catalog number: 11-500)
7. Fetal bovine serum (FBS) (Corning, catalog number: 35-011-CV)
8. Plasmid pcDNA3.1-corin-V5
*
Note: This plasmid was generated by inserting a cDNA encoding human corin into a pcDNA3.1/V5 vector (Thermo Fisher, catalog number: K480001), as published previously (Knappe et al., 2003).
*
9. Plasmid pcDNA3.1-prothrombin-V5
*
Note: This plasmid was generated by inserting a cDNA encoding human prothrombin into a pcDNA3.1/V5 vector (Thermo Fisher, catalog number: K480001), as published previously (Wang et al., 2018).
*
10. OPTI-MEM (Thermo Fisher, catalog number: 11058021)
11. Fugene transfection reagent (Promega, catalog number: E2311)
12. Phosphate buffered saline (PBS), 10x (Affymetrix, catalog number: 75889)
13. Trypsin-EDTA solution (Corning, catalog number: 25053CI)
14. Geneticin (Fisher Scientific, catalog number: BP6731)
15. Glycine  (Research Products International (RPI), catalog number:  G36050)
16. Tris-base (Fisher Scientific, catalog number: 502-13-709)
17. Sodium chloride (RPI, catalog number: S23020)
18. Nonidet P-40 (Affymetrix, catalog number: 19628)
19. Protease inhibitor cocktail (Sigma-Aldrich, catalog number: P8340)
20. Sodium dodecyl sulfate (SDS) (Fisher Scientific, catalog number: BP166500)
21. SDS-PAGE protein sample buffer (2x) ( Bio-Rad,  catalog number:  1610737)
22. β-mercaptoethanol (Fisher Scientific, catalog number: BP176-100)
23. Pre-stained protein ladder (Thermo Fisher, catalog number: 26616)
24. Tris-Glycine gel (4-20%) (Thermo Fisher, catalog number: XP04200BOX)
25. Methanol (any brand, ACS grade)
26. Polyvinylidene difluoride membrane for Western blotting (Millipore, catalog number: IPVH00010)
27. Tris-buffered saline and Tween 20 (TBST), 20x (Santa Cruz Biotechnology, catalog number: sc-362311)
28. Non-fat milk (Bio-Rad, catalog number: 1706404)
29. Horseradish peroxidase (HRP) conjugated anti-V5 antibody (Thermo Fisher, catalog number: R96125)
30. 1-deoxynojirimycin (DNJ) (Alfa Aesar, catalog number: J62602)
31. Cell lysis buffer (see Recipes)
32. SDS-PAGE buffer (see Recipes)
33. Western blot transfer buffer (see Recipes)
34. Western blot blocking buffer (see Recipes)

## Equipment

1. Pipetman P20 (Gilson, catalog number: F144801)
2. Pipetman P200 (Gilson, catalog number: F123601)
3. Pipetman P1000 (Gilson, catalog number: F123602)
4. Humidified cell culture incubator (any brand)
5. Microcentrifuge (Thermo Fisher, catalog number: 75002446)
6. Benchtop rocker (any brand)
7. Heating block up to 100 °C (any brand)
8. Mini gel apparatus (Thermo Fisher, catalog number: A25977)
9. Gel transfer apparatus (Bio-Rad, catalog number: 1660827EDU)
10. Power Supply (Bio-Rad, catalog number: 1645052)
11. Ice bucket (any brand)

## Procedure

- Generation of HEK293 cells stably expressing recombinant glycoproteins (corin or prothrombin)
  1. Seed HEK293 cells in a 6-well plate (3 x 10^5^ cells per well) with Dulbecco’s Modified Eagle’s medium (DMEM, 2 ml per well) containing 10% FBS.
  2. Culture the cells at 37 °C in a humidified incubator to 70-80% of confluency (24 h).
  3. Transfect HEK293 cells with the plasmid (2 μg, expressing human corin or prothrombin containing a C-terminal V5 tag) using OPTI-MEM medium (85 μl) and Fugene transfection reagent (6 μl), according to the manufacturer’s protocol.
  4. Culture the cells at 37 °C for 2 days.
  5. Remove the medium and wash the cells once with PBS (5 ml per well).
  6. Treat the cells with trypsin-EDTA solution (1 ml) at 37 °C for 1-2 min. Check the cells under a microscope to ensure that > 70% of cells are detached. (HEK293 cells attached to the culture plate are flat, whereas the detached cells are round.)
  7. Tap the culture plate gently so that > 90% of cells are detached. Stop the trypsin activity by adding fresh DMEM with 10% FBS (1 ml per well).
  8. Transfer the transfected HEK293 cells into 100 mm dishes (2.2 x 10^6^ cells per dish) with 10 ml of DMEM containing 10% FBS and geneticin (G418, 400 μg/ml), an antibiotic commonly used for the selection of stably transfected mammalian cells. The concentration of G418 was based on the published information and the laboratory’s previous experience.
  9. Culture the transfected cells and replace the culture medium with fresh medium every other day, for 2 weeks. No need to passage cells during this time.
  10. Maintain the remaining G418 resistant cells for the following experiments.
- Verification of recombinant protein expression in stably transfected HEK293 cells
  1. Seed stably transfected HEK293 cells in a 6-well plate (3 x 10^5^ cells in 2 ml DMEM per well) and culture the cells at 37 °C to 90% of confluency (36 h).
  2. Gently remove the medium by suction and wash the cells once with PBS (pre-chilled on ice, 1 ml per well).
  3. Detach the cells by flushing with 1 ml of pre-chilled PBS using a pipet until all cells are detached.
  4. Transfer the cells to a 1.5 ml microcentrifuge tube.
  5. Spin down the cells with a microcentrifuge (900 *× g* at 4 °C for 5 min) and discard the supernatant.
  6. Lyse the pelleted cells in a cell lysis buffer (50 μl) at 4 °C for 30 min (tap the tube every 5 min to ensure the cells were completely lysed).
  7. Centrifuge the lysis mixture at 16,200 *× g*, 4 °C, for 10 min.
  8. Collect the supernatant (cell lysate, 50 μl) and discard the insoluble cellular debris.
  9. Denature the cell lysate with SDS-PAGE sample buffer (2x, 50 μl) containing 5% β-mercaptoethanol at 95 °C for 5 min and cool down the protein sample to room temperature.
  10. Confirm the expression of recombinant glycoproteins by SDS-PAGE and Western blotting with the conditions indicated in the table below.
    **Table:** 

Step	Condition	Time
SDS-PAGE	160 V, room temperature	1.5 h
Protein transfer	100 V, 4 °C	1 h
Incubation with blocking buffer (see Recipes)	4 °C	1 h
Western blotting	Anti-V5-HRP antibody (1:5,000 in blocking buffer), room temperature for 2 h or 4 °C overnight (o/n), based on pilot experiments.	2 h or o/n
- Inhibition of α-glucosidases I and II by DNJ
  1. Seed HEK293 cells stably expressing recombinant proteins (corin or prothrombin) in a 6-well plate or a 100 mm dish, depending on the further analysis, with DMEM containing 10% FBS and G418 (400 μg/ml) (optional but recommended).
  2. Culture the cells to 70-80% of confluency.
  3. Replace the medium with fresh DMEM containing 10% FBS, G418 (400 μg/ml) (optional), and DNJ (2 mM) to block the activity of α-glucosidases I and II.
  4. Culture the cells at 37 °C for 24-48 h.
  5. Analyze the glycoproteins of interest in the cells and the conditioned medium, depending on research purposes (see Notes 4-6 below).

## Notes

1. This protocol was used for studying the calnexin-assisted folding and extracellular expression of recombinant corin (a cell surface protein) and prothrombin (a secreted protein) (Wang *et al.*, 2018).
2. The expression plasmids used in transfection (in Step A2) and the verification (in Procedure B) should be modified based on glycoproteins of interest.
3. The time of DNJ inhibition (in Steps C3-C4) can be adjusted depending on specific cell types and glycoproteins of interest. This may require pilot experiments.
4. In general, DNJ inhibition blocks the calnexin- and/or calrecticulin-assisted glycoprotein folding, resulting in protein retention by ER chaperones such as binding immunoglobulin protein (BiP), which can be further verified by co-immunoprecipitation with ER proteins and/or co-immune staining with ER markers (Wang *et al.*, 2018).
5. For glycoproteins that are expressed on the cell surface, *e.g.*, corin, DNJ inhibition is expected to impair intracellular trafficking and reduce cell surface expression, which can be verified by cell surface protein labeling and/or immune staining (Wang *et al.*, 2018).
6. For glycoproteins that are secreted, *e.g.*, prothrombin, DNJ inhibition is expected to reduce their extracellular secretion, which can be verified by Western blotting and/or ELISA (Wang *et al.*, 2018).

## Data analysis

**Figure 3.**
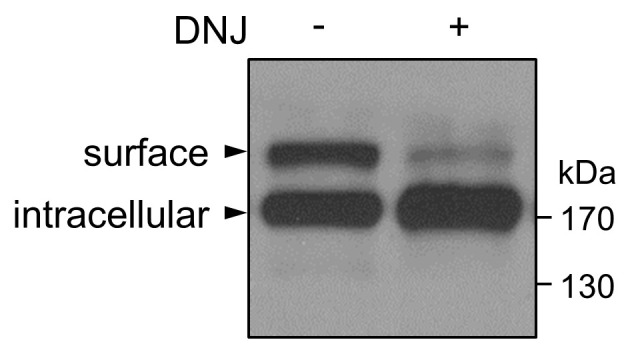
Western blotting analysis of corin protein . Transfected HEK293 cells expressing human corin were cultured in the absence (-) or presence (+) of DNJ (2 mM). After 48 h, the cells were lysed and analyzed by Western blotting using an anti-V5 antibody. In the cells treated with DNJ, corin expression on the cell surface, but not inside the cell, was decreased. In previous studies with trypsin digestion, cell surface protein labeling, and glycosidase endo H digestion, the top and lower bands were shown to represent cell surface and intracellular corin proteins, respectively (Chen *et al.*, 2015; Wang *et al.*, 2018).

Depending on proteins of interest and the experimental design, enhanced ER chaperone binding, reduced cell surface expression, and reduced protein secretion can be analyzed by methods described in Notes 4-6. Western blotting can be analyzed qualitatively with visual inspection and/or quantitatively with densitometry. Immune staining can be analyzed with ImageJ, a Java-based image processing program (the National Institutes of Health) (Wang *et al.*, 2018). As an example, DNJ treatment reduced corin expression on the surface of HEK293 cells, which was analyzed by Western blotting (Figure 3) (Wang *et al.*, 2018).

## Recipes

1. Cell lysis buffer
  50 mM Tris-base (pH 8.0)
  150 mM sodium chloride
  1% Nonidet P-40 (vol/vol)
  1% protease inhibitor cocktail (vol/vol)
2. SDS-PAGE buffer
  25 mM Tris-base
  250 mM glycine
  3.5 mM SDS
3. Western blot transfer buffer
  25 mM Tris-base
  190 mM glycine
  20% methanol (vol/vol)
4. Western blot blocking buffer
  5% non-fat milk in TBST (w/w)
